# The efficacy of low-dose aspirin in pregnancy among women in malaria-endemic countries

**DOI:** 10.1186/s12884-022-04652-9

**Published:** 2022-04-10

**Authors:** Melissa Bauserman, Sequoia I. Leuba, Jennifer Hemingway-Foday, Tracy L. Nolen, Janet Moore, Elizabeth M. McClure, Adrien Lokangaka, Antoinette Tsehfu, Jackie Patterson, Edward A. Liechty, Fabian Esamai, Waldemar A. Carlo, Elwyn Chomba, Robert L. Goldenberg, Sarah Saleem, Saleem Jessani, Marion Koso-Thomas, Matthew Hoffman, Richard J. Derman, Steven R. Meshnick, Carl L. Bose

**Affiliations:** 1 University of North Carolina at Chapel Hill, Chapel Hill, Chapel Hill, NC USA; 2 RTI International, Durham, NC USA; 3 Kinshasa School of Public Health, Kinshasa, Democratic Republic of Congo; 4 Indiana University School of Medicine, University of Indiana, Indianapolis, IN USA; 5 Moi University School of Medicine, Eldoret, Kenya; 6 University of Alabama at Birmingham, Birmingham, AL USA; 7 University Teaching Hospital, Lusaka, Zambia; 8 Department of Obstetrics and Gynecology, Columbia University School of Medicine, New York, NY USA; 9 Aga Khan University, Karachi, Pakistan; 10 Eunice Kennedy Shriver National Institute of Child Health and Human Development, Bethesda, MD USA; 11 Department of Obstetrics and Gynecology, Christiana Care, Newark, DE USA; 12 Thomas Jefferson University, Philadelphia, USA

**Keywords:** Malaria, Pregnancy, Premature birth, Perinatal mortality

## Abstract

**Background:**

Low dose aspirin (LDA) is an effective strategy to reduce preterm birth. However, LDA might have differential effects globally, based on the etiology of preterm birth. In some regions, malaria in pregnancy could be an important modifier of LDA on birth outcomes and anemia.

**Methods:**

This is a sub-study of the ASPIRIN trial, a multi-national, randomized, placebo controlled trial evaluating LDA effect on preterm birth. We enrolled a convenience sample of women in the ASPIRIN trial from the Democratic Republic of Congo (DRC), Kenya and Zambia. We used quantitative polymerase chain reaction to detect malaria. We calculated crude prevalence proportion ratios (PRs) for LDA by malaria for outcomes, and regression modelling to evaluate effect measure modification. We evaluated hemoglobin in late pregnancy based on malaria infection in early pregnancy.

**Results:**

One thousand four hundred forty-six women were analyzed, with a malaria prevalence of 63% in the DRC site, 38% in the Kenya site, and 6% in the Zambia site. Preterm birth occurred in 83 (LDA) and 90 (placebo) women, (PR 0.92, 95% CI 0.70, 1.22), without interaction between LDA and malaria (*p* = 0.75). Perinatal mortality occurred in 41 (LDA) and 43 (placebo) pregnancies, (PR 0.95, 95% CI 0.63, 1.44), with an interaction between malaria and LDA (*p* = 0.014). Hemoglobin was similar by malaria and LDA status.

**Conclusions:**

Malaria in early pregnancy did not modify the effects of LDA on preterm birth, but modified the effect of LDA on perinatal mortality. This effect measure modification deserves continued study as LDA is used in malaria endemic regions.

## Background

Preterm birth is a major cause of neonatal mortality, and infants born in low- and middle-income countries (LMICs) are particularly vulnerable. Recent trials demonstrate that low dose aspirin (LDA) is an effective strategy to reduce preterm birth [1–3]. However, LDA might have differential effects in global regions, based on the etiology of preterm birth. The *Eunice Kennedy Shriver* National Institute for Child Health and Human Development (NICHD) Global Network for Women’s and Children’s Health Research (GN) recently completed the ASPIRIN trial, a multi-country trial of the effects of LDA on preterm birth [3, 4]. While, this study was not powered to investigate efficacy in individual sites, site-specific data did not demonstrate a reduction in the risk of preterm birth for LDA in the Democratic Republic of the Congo (DRC) [3]. One feature that distinguished this site from others in the ASPIRIN trial was high malaria prevalence.

Malaria infections during pregnancy are an important cause of preterm birth in many LMICs, especially sub-Saharan African countries where malaria infection in pregnancy is common [5]. Malaria is associated with up to 36% of all preterm or low birth weight (LBW) infants born in malaria endemic areas [6]. Malaria treatment or prophylaxis in the latter half of pregnancy improves outcomes [7]. Studies also suggest that malaria may exert deleterious effects early in pregnancy that lead to preterm birth, stillbirth, pregnancy loss, anemia or other adverse pregnancy outcomes [7–9]. Malaria infections during early pregnancy are associated with abnormal development of the placental circulation that may result in hypertensive disorders, intrauterine growth restriction (IUGR) and prematurity [5]. Early malaria infections may cause both local and systemic inflammation leading to poor placentation that leads to these adverse pregnancy outcomes.

LDA is thought to reduce inflammation and thrombosis that leads to placental dysfunction, preterm birth, preeclampsia and IUGR [10, 11]. Malaria could be an important effect modifier of the relationship between LDA and preterm birth in malaria-endemic regions. For example, malaria may reduce the beneficial effects of LDA by increasing inflammation. Conversely, LDA may reduce the adverse impact of malaria in early pregnancy by reducing placental inflammation caused by malaria.

LDA treatment to prevent preterm birth has important public health implications, and might be adopted in LMICs, including those countries in malaria-endemic regions. Therefore, there is an urgent need to understand the relationships between LDA and malaria in early pregnancy on maternal and neonatal health. The objectives of this paper were to investigate if LDA has a differential effect in areas where malaria is prevalent and determine whether malaria infection interacts with the effects of LDA on pregnancy outcomes. Furthermore, we also investigated the relationships between malaria, LDA and anemia in pregnancy to determine if a potential effect of malaria was mediated through anemia, a common morbidity of the disease.

## Methods

This study was a sub-study of the ASPIRIN (Aspirin Supplementation for Pregnancy Indicated Risk Reduction In Nulliparas) trial [4]. The ASPIRIN trial was a multi-national, randomized, double-masked, placebo controlled trial to evaluate the effects of LDA (81 mg daily) in nulliparous women on the outcome of preterm birth (birth before 37 weeks gestational age). Across 8 research sites in LMICs, women were randomized in a 1:1 ratio to receive LDA (treatment) or an identically-appearing placebo (control).

Pregnant women were eligible for the ASPIRIN trial if they were nulliparous, between 14–40 years of age, had pregnancies characterized by a gestational age between 6 weeks and 0 days and 13 weeks and 6 days (confirmed by ultrasound), blood pressure < 140/90 mmHg, hemoglobin > 7.0 g/dL, a live singleton fetus, and absence of fetal anomaly. Women were excluded who had allergy or contraindication to aspirin, previous aspirin use for more than 7 days, a history of ≥ 2 first-trimester losses, or the presence of diabetes or hypertension [4].

For the sub-study, we enrolled a convenience sample of women participating in the ASPIRIN trial from 4 of the 8 GN sites. The four sites were chosen based on the potential for malaria affecting the pregnancies in the study regions, and represented a range of malaria endemicity (high and low). These 4 sites were located in the DRC (North and South Ubangi Provinces), Kenya (Western region), Pakistan (near the city of Karachi), and Zambia (south and east of the capital city of Lusaka).

Women enrolled in the malaria sub-study had blood collected at times coincident with blood collection in the ASPIRIN trial. Blood was collected at enrollment (6–13 6/7 weeks gestation) and during the third trimester (between 26–30 weeks gestation), using dried blood spot techniques. All blood spot cards were shipped to a central laboratory, the Meshnick Laboratory at the University of North Carolina (U.S.) for analyses. The PCR results were performed in batches, in most cases, after the conclusion of pregnancies, therefore the PCR research results were not available to guide treatment decisions during pregnancy. Rapid diagnostic tests (RDTs) were also performed for women enrolled at the Pakistan, Zambia and Kenya sites, and women were treated according to local standards based on the results. No RDTs were performed at the DRC site. Each sample was tested in duplicate for *P. falciparum* lactate dehydrogenase (pfldh) by quantitative polymerase chain reaction (qPCR). Because malaria parasites were not detected in the samples from Pakistan, the women enrolled from the Pakistani site were excluded from subsequent analyses.

We defined primary and secondary efficacy outcomes as specified in the ASPIRIN trial. Therefore, preterm delivery was defined as delivery after 20 weeks, but before 37 0/7 weeks gestation. We defined hypertensive disorders to include pre-eclampsia, eclampsia, and gestational hypertension. We defined small for gestational age as a birth weight less than 10% of the INTERGROWTH-21^st^ growth standard.[12] We defined perinatal mortality as stillbirths and deaths in the perinatal period from 20 weeks gestation to 7 days post-partum. Additionally, we used a priori specifications for other maternal outcomes of interest, such as maternal vaginal bleeding, antepartum/postpartum hemorrhage, maternal mortality and a combined category of preterm and hypertensive disorders. We used a priori defined specifications for other fetal outcomes of interest, including preterm delivery < 34 weeks, birthweight < 2500 g, birthweight < 1500 g and stillbirth. We also defined malaria in late pregnancy as an infection in women for whom we collected a secondary blood sample between 26–30 weeks that was positive for malaria by qPCR.

We performed descriptive statistics for bivariate comparisons. Crude prevalence proportion ratios (PRs) were calculated for LDA treatment by *P. falciparum* infection in the first trimester by each variable using stratified tables for all sites and for each site individually. We chose to present prevalence proportion ratios instead of incidence ratios since our enrollment occurred after conception, and therefore true incidence of adverse pregnancy outcomes (outcomes from conception to end of pregnancy) could not be accurately determined. Poisson models were developed to evaluate effect measure modification of malaria on the effect of LDA in regards to maternal and fetal outcomes of interest with terms included for treatment, malaria in the first trimester, site, and the interaction term of malaria in the first trimester and LDA treatment. Similar models were constructed separately for each site to estimate the prevalence ratio of preterm birth and perinatal mortality with terms included for treatment, malaria in the first trimester, and the interaction term of malaria in the first trimester and LDA treatment.

We used prevalence ratios (PRs) to investigate the relationship between early pregnancy malaria status and late pregnancy hemoglobin levels. Hemoglobin levels were measured by point of care testing devices in the third trimester (between 26–30 weeks). We used multiple cutoffs for hemoglobin levels (≤ 8 g/dL, ≤ 9 g/dL, ≤ 10 g/dL, and ≤ 11 g/dL) to assess whether malaria in the first trimester was an effect measure modifier for the relationship between LDA treatment and hemoglobin levels in late pregnancy. Comparisons were limited to observations without missing data for each variable. All analyses were performed using the R statistical platform (version 4.0.2) and SAS version 9.4 (SAS Institute, Cary, NC, USA).

This study was approved by the relevant ethics committees at the institutions conducting the study at each site prior to the initiation of study activities. The study was also approved by the ethics committees at the partner U.S.-based institutions (University of North Carolina at Chapel Hill, Columbia University, University of Alabama at Birmingham and Indiana University) and by RTI International, the data coordinating center. All women provided informed consent prior to their participation in the sub-study. The ASPIRIN trial was registered in clinicaltrials.gov (NCT02409680).

## Results

**Fig. 1:**
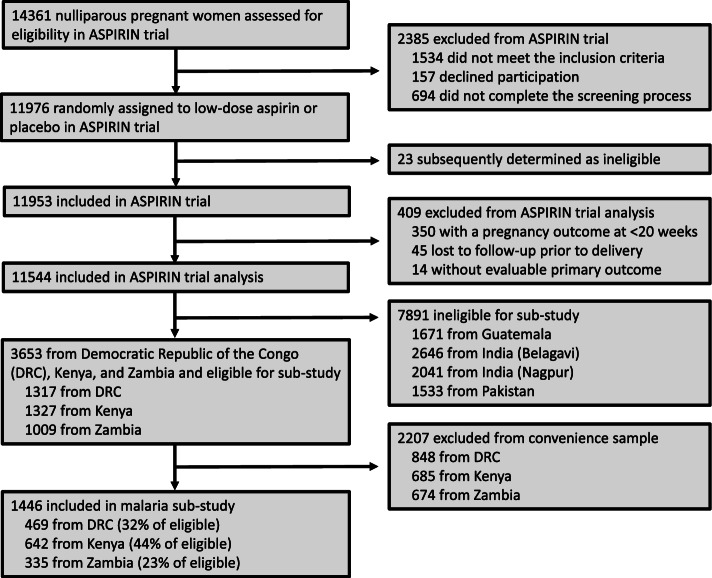
Study Participants

We included 1,446 pregnant women in our analyses; 469 (32%) from DRC, 642 (44%) from Kenya, and 335 (23%) from Zambia (Fig. 1).

The women in the DRC site had the highest prevalence of malaria in early pregnancy (297/469, 63%), followed by the women in the Kenya site (244/642, 38%), then the Zambia site (21/335, 6%). Half of the women (n = 724, 50%) were randomized to the LDA (treatment) arm. Of the women who received LDA, 39% (279/724) had malaria in early pregnancy, equal to 39% of women (283/722) with malaria in early pregnancy who were randomized to placebo (Table 1).

**Table 1:** Baseline characteristics of study population by treatment arm and malaria status in early pregnancy

**Variable**	**ALL CALL COUNTRIES**			
	**LDA (*N* = 724)**		**PLACEBO (*N* = 722)**	
	**Malaria negative**	**Malaria positive**	**Malaria negative**	**Malaria positive**
**Randomized, N (%)**	445 (61)	279 (39)	439 (61)	283 (39)
**Maternal age (years), N (%)**				
< 20	225 (50·6)	209 (74·9)	250 (56·9)	206 (72·8)
20–29	214 (48·1)	69 (24·7)	185 (42·1)	74 (26·1)
> 29	6 (1·3)	1 (0·4)	4 (0·9)	3 (1·1)
Median (P25, P75)	19·0 (18·0, 22·0)	18·0 (17·0, 19·5)	19·0 (18·0, 21·0)	18·0 (17·0, 20·0)
**Projected gestational age at enrollment (weeks, days), N (%)**				
6, 0 – 7, 6	63 (14·2)	30 (10·8)	72 (16·4)	26 (9·2)
8, 0 – 9, 6	134 (30·1)	75 (26·9)	114 (26·0)	76 (26·9)
10, 0 – 10, 6	66 (14·8)	33 (11·8)	57 (13·0)	31 (11·0)
11, 0 – 11,6	47 (10·6)	48 (17·2)	66 (15·0)	54 (19·1)
12, 0 – 13, 6	135 (30·3)	93 (33·3)	130 (29·6)	96 (33·9)
Median (P25, P75)	10·3 (8·7, 12·3)	11·0 (9·1, 12·4)	10·6 (8·4, 12·1)	11·1 (9·0, 12·4)
**Maternal education, N (%)**				
No formal	20 (4·5)	25 (9·0)	19 (4·3)	24 (8·5)
Primary	58 (13·0)	84 (30·1)	60 (13·7)	97 (34·3)
Secondary	334 (75·1)	158 (56·6)	327 (74·5)	156 (55·1)
University +	33 (7·4)	12 (4·3)	33 (7·5)	6 (2·1)
**Maternal height (cm), N**				
Mean (StdDev)	157·0 (7·5)	155·6 (8·0)	157·0 (7·4)	155·2 (7·9)
Median (P25, P75)	158·0 (152·5, 162·0)	156·0 (150·2, 161·0)	157·0 (152·5, 161·9)	155·0 (150·0, 160·0)
**Maternal weight (kg), N**				
Mean (StdDev)	55·0 (8·1)	53·0 (7·4)	55·0 (8·3)	52·3 (7·5)
Median (P25, P75)	54·0 (50·0, 59·0)	52·2 (48·0, 58·0)	54·0 (50·0, 60·0)	52·0 (47·0, 56·8)
**Maternal BMI (kg/m2), N**				
Mean (StdDev)	22·4 (3·4)	22·0 (3·1)	22·3 (3·4)	21·8 (3·1)
Median (P25, P75)	21·1 (20·0, 24·0)	21·7 (19·7, 23·4)	21·9 (20·0, 24·1)	21.5 (19·5, 23·3)
**Antenatal care visits, N**				
Mean (StdDev)	4·2 (1·1)	3·9 (1·3)	4·2 (1·1)	3·9 (1·2)
Median (P25, P75)	4·0 (4·0, 5·0)	4·0 (3·0, 5·0)	4·0 (4·0, 5·0)	4·0 (3·0, 5·0)
**Delivery attendant, N (%)**				
Physician	24 (5·4)	9 (3·2)	18 (4·1)	9 (3·2)
Nurse/nurse midwife	391 (87·9)	241 (86·4)	383 (87·2)	239 (84·5)
Traditional birth attendant	19 (4·3)	22 (7·9)	23 (5·2)	30 (10·6)
Family/Self/Other	11 (2·5)	7 (2·5)	15 (3·4)	5 (1·8)
**Delivery location, N (%)**				
Hospital	113 (25·4)	58 (20·8)	122 (27·8)	46 (16·3)
Clinic/health center	296 (66·5)	180 (64·5)	270 (61·5)	194 (68·6)
Home/Other	36 (8·1)	41 (14·7)	47 (10·7)	43 (15·2)
**Delivery mode, N (%)**				
Vaginal	426 (95·7)	271 (97·1)	422 (96·1)	276 (97·5)
C-section	19 (4·3)	8 (2·9)	17 (3·9)	7 (2·5)
**Site, N (%)**				
DRC	87 (19·6)	140 (50·2)	85 (19·4)	157 (55·5)
Kenya	199 (44·7)	130 (46·6)	199 (45·3)	114 (40·3)
Zambia	159 (35·7)	9 (3·2)	155 (35·3)	12 (4·2)

Baseline characteristics were similar among women who received LDA vs placebo, and among women who had malaria in early pregnancy vs those who did not, in terms of gestational age at enrollment, maternal height, weight, body mass index (BMI), antenatal care visits, delivery attendant, delivery location and mode of delivery (Table 1). Women with malaria in early pregnancy trended toward younger maternal ages and fewer years of formal education. The majority of all mothers had deliveries attended by nurses/midwives, delivered in clinics or health centers and had vaginal deliveries. The median number of antenatal care (ANC) visits was four.

Preterm birth occurred in 83 women in the LDA group and 90 women in the placebo group (PR 0.92, 95% CI 0.70, 1.22) (Table 2).

**Table 2:** Primary and secondary efficacy outcomes based on infection with malaria in early pregnancy

Variable	Overall		Malaria Negative		Malaria Positive		Aspirin – Malaria Interaction *p*-value
	**n/N (%)**	**PR (95% CI)**	**n/N (%)**	**PR (95% CI)**	**n/N (%)**	**PR (95% CI)**	
**Primary Outcome**							
Preterm delivery							
Aspirin	83/724 (11)	0·92 (0·70, 1·22)	38/445 (9)	0·89 (0·59, 1·36)	45/279 (16)	0·95 (0·66, 1·38)	0.75
Placebo	90/722 (12)		42/439 (10)		48/283 (17)		
Hypertensive disorders							
Aspirin	10/724 (1)	1·11 (0·45, 2·71)	8/445 (2)	0·88 (0·34, 2·25)	2/279 (1)	5·07 (0·24, 105·17)	–
Placebo	9/722 (1)		9/439 (2)		0/283 (0)		
Small for gestational age							
Aspirin	89/686 (13)	1·11 (0·84, 1·48)	58/421 (14)	1·21 (0·84, 1·73)	31/265 (12)	0·97 (0·61, 1·54)	0.62
Placebo	78/668 (12)		46/403 (11)		32/265 (12)		
Perinatal mortality							
Aspirin	41/724 (6)	0·95 (0·63, 1·44)	16/445 (4)	0·56 (0·31, 1·03)	25/279 (9)	1·69 (0·91, 3·14)	0.014
Placebo	43/722 (6)		28/439 (6)		15/283 (5)		
**Other Maternal Outcomes of Interest**							
Vaginal bleeding							
Aspirin	12/713 (2)	0·86 (0·40, 1·85)	11/437 (3)	0·91 (0·41, 2·05)	1/276 (0)	0·51 (0·05, 5·56)	0.64
Placebo	14/715 (2)		12/435 (3)		2/280 (1)		
Antepartum hemorrhage							
Aspirin	4/721 (1)	8·95 (0·48, 165·94)	4/442 (1)	8·86 (0·48, 164·04)	0/279 (0)	1·01 (0·02, 50·76)	–
Placebo	0/717 (0)		0/435 (0)		0/282 (0)		
Postpartum hemorrhage							
Aspirin	6/724 (1)	2·99 (0·61, 14·77)	2/445 (0)	4·93 (0·24, 102·46)	4/279 (1)	2·03 (0·37, 10·99)	–
Placebo	2/722 (0)		0/439 (0)		2/283 (0)		
Maternal mortality through 42 days							
Aspirin	0/724 (0)	0·33 (0·02, 8·15)	0/445 (0)	0·33 (0·02, 8·05)	0/279 (0)	1·01 (0·02, 50·94)	–
Placebo	1/722 (0)		1/439 (0)		0/283 (0)		
Preterm and hypertensive disorders							
Aspirin	1/724 (0)	2·99 (0·12, 73·32)	0/445 (0)	0·99 (0·02, 49·61)	1/279 (0)	3·04 (0·12, 74·38)	–
Placebo	0/722 (0)		0/439 (0)		0/283 (0)		
**Other Fetal Outcomes of Interest**							
Preterm < 34 weeks of pregnancy							
Aspirin	30/724 (4)	0·88 (0·54, 1·42)	15/445 (3)	0·74 (0·38, 1·43)	15/279 (5)	1·09 (0·53, 2·21)	0.40
Placebo	34/722 (5)		20/439 (5)		14/283 (5)		
Birth weight < 2500 g							
Aspirin	76/715(11)	0·94 (0·70, 1·27)	37/439 (8)	0·91 (0·59, 1·39)	39/276 (14)	0·98 (0·65, 1·47)	0.69
Placebo	80/708 (11)		40/432 (9)		40/276 (15)		
Birth weight < 1500 g							
Aspirin	9/715 (1)	0·69 (0·29, 1·59)	1/439 (0)	0·10 (0·01, 0·77)	8/276 (3)	2·67 (0·71, 9·95)	0.007
Placebo	13/708 (2)		10/432 (2)		3/276 (1)		
Stillbirth							
Aspirin	14/718 (2)	0·87 (0·43, 1·76)	8/442 (2)	0·87 (0·34, 2·23)	6/276 (2)	0·87 (0·30, 2·55)	0.99
Placebo	16/712 (2)		9/432 (2)		7/280 (3)		
Malaria in late pregnancy (26–30 weeks)							
Aspirin	92/296 (31)	1·13 (0·88, 1·45)	42/177 (24)	1·42 (0·93, 2·15)	50/119 (42)	0·94 (0·70, 1·26)	0.15
Placebo	83/301 (28)		31/185 (17)		52/116 (45)		

The association between LDA and preterm birth was similar among the malaria negative (PR 0.89, 95% CI 0.59, 1.36) and the malaria positive group (PR 0.95, 95% CI 0.66, 1.38, *p* = 0.75). Perinatal mortality occurred in 41 pregnancies in the LDA group and 43 pregnancies in the placebo group (PR 0.95, 95% CI 0.63, 1.44). The association between LDA and perinatal mortality varied between the malaria negative (PR 0.56, 95% CI 0.31, 1.03) and the malaria positive group (PR 1.69, 95% CI 0.91, 3.14), as the model indicated interaction between LDA treatment and malaria infection for perinatal mortality, (*p* = 0.014).

Malaria in late pregnancy was present in 92 pregnancies in the LDA group and 83 pregnancies in the placebo group (PR 1.13, 95% CI 0.88, 1.45) (Table 2). The model did not show a significant interaction between LDA treatment and early malaria infection on late malaria status (*p* = 0.15). Outcomes of hypertensive disorders, small for gestational age, vaginal bleeding, antepartum hemorrhage, postpartum hemorrhage, maternal mortality, and the combined outcome of preterm and hypertensive disorders occurred infrequently among the groups. The protective effect of LDA on very low birth weight (< 1500 g) is observed in the malaria negative group but not in the malaria positive group. No evidence of an interaction between LDA and malaria status was observed for any other secondary neonatal outcomes of interest (i.e., interaction terms in model-based analyses were not significant and PRs numerically do not appear to differ).

Preterm birth and perinatal mortality occurred at different frequencies in each research site (Table 3).

**Table 3:** Preterm birth and perinatal mortality outcomes, by site

Outcome	Overall		Malaria Negative		Malaria positive		Aspirin – Malaria Interaction *p*-value
	**n/N (%)**	**PR (95% CI)**	**N w/o outcome**	**PR (95% CI)**	**N w/o outcome**	**PR (95% CI)**	
***DRC DRC***							
Preterm Delivery							
Aspirin	44/227 (19)	0·98 (0·68, 1·41)	14/87 (16)	1·05 (0·53, 2·10)	30/140 (21)	0·96 (0·62, 1·48)	0.84
Placebo	48/242 (20)		13/85 (15)		35/157 (22)		
Perinatal Mortality							
Aspirin	18/227 (8)	1·37 (0·70, 2·69)	3/87 (3)	0·42 (0·11, 1·57)	15/140 (11)	2·40 (1·01, 5·72)	0.035
Placebo	14/242 (6)		7/85 (8)		7/157 (4)		
**Kenya**							
Preterm Delivery							
Aspirin	29/329 (9)	0·95 (0·58, 1·55)	15/199 (8)	0·94 (0·48, 1·84)	14/130 (11)	0·94 (0·46, 1·92)	0.99
Placebo	29/313 (9)		16/199 (8)		13/114 (11)		
Perinatal Mortality							
Aspirin	17/329 (5)	0·95 (0·49, 1·83)	7/199 (4)	0·78 (0·30, 2·05)	10/130 (8)	1·10 (0·45, 2·68)	0.62
Placebo	17/313 (5)		9/199 (5)		8/114 (7)		
**Zambia**							
Preterm Delivery							
Aspirin	10/168 (6)	0·76 (0·34, 1·70)	9/159 (6)	0·67 (0·30, 1·53)	1/9 (11)	–	–
Placebo	13/167 (8)		13/155 (8)		0/12 (0)		
Perinatal Mortality							
Aspirin	6/168 (4)	0·50 (0·19, 1·29)	6/159 (4)	0·49 (0·19, 1·27)	0/9 (0)	–	1.00
Placebo	12/167 (7)		12/155 (8)		0/12 (0)		

Notes: PR is prevalence ratio

PR was not calculated for cells in which no participants experienced the outcome

In the DRC, 92/469 (20%) pregnancies ended in preterm birth, compared to 58/642 (9%) in Kenya and 23/335 (7%) in Zambia. Perinatal mortality was observed with an incidence of 7% (32/469) in the DRC, 5% (34/642) in Kenya, and 5% (18/335) in Zambia. Effect measure modification was observed in site specific analyses only for perinatal mortality for DRC (*p* = 0.035).

We report hemoglobin levels in late pregnancy based on malaria infection in early pregnancy (Table 4).

**Table 4:** Hemoglobin level, by malaria infection and aspirin exposure

**Hemoglobin cut-point**	**Overall**		**Malaria Negative**		**Malaria positive**	
	**N (w/ and w/o outcome)**	**PR (95% CI)**	**N (w/ and w/o outcome)**	**PR (95% CI)**	**N (w/ and w/o outcome)**	**PR (95% CI)**
Hemoglobin ≤ 8 g/dL						
Aspirin	11/618	0·91 (0·04, 2·04)	3/376	0·58 (0·14, 2·43)	8/242	1·16 (0·43, 3·14)
Placebo	12/610		5/364		7/246	
Hemoglobin ≤ 9 g/dL						
Aspirin	43/586	1·06 (0·70, 1·58)	10/369	0·57 (0·27, 1·23)	33/217	1·34 (0·82, 2·18)
Placebo	42/580		17/352		25/228	
Hemoglobin ≤ 10 g/dL						
Aspirin	122/507	1·01 (0·80, 1·26)	49/330	1·04 (0·71, 1·51)	73/177	1·00 (0·76, 1·31)
Placebo	120/502		46/323		74/179	
Hemoglobin ≤ 11 g/dL						
Aspirin	266/363	1·03 (0·90, 1·17)	128/251	1·07 (0·87, 1·31)	138/112	1·00 (0·86, 1·18)
Placebo	256/366		117/252		139/114	

Note: Hemoglobin measured between 26–30 weeks. Malaria in early pregnancy measured between 6–13 6/7 weeks gestation

**Fig. 2:**
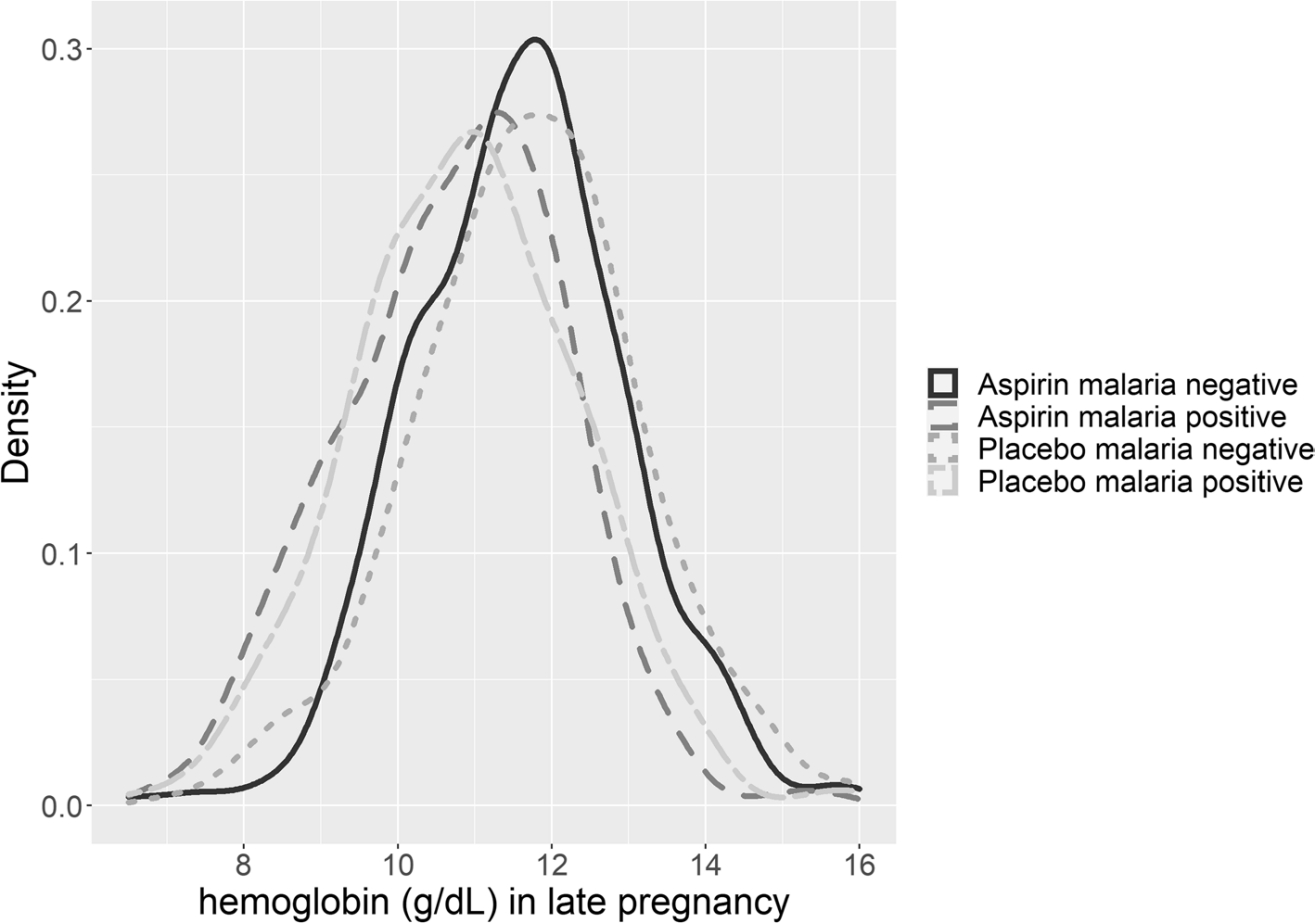
Hemoglobin level in late pregnancy (weeks 26–30), by malaria status in early pregnancy and aspirin exposure

Hemoglobin levels in late pregnancy appeared to be similar among women with malaria positive and women that were malaria negative, and LDA treated and untreated groups (Fig. 2).

## Discussion

Our study investigated the potential interaction between LDA and malaria in early pregnancy. We found that malaria in early pregnancy did not modify the effects of LDA on the risk of preterm birth. However, we did observe effect measure modification between LDA and malaria on perinatal mortality. Women without malaria in early pregnancy had lower rates of perinatal mortality when given LDA versus placebo, but this protective effect was not observed for women with malaria in early pregnancy. Third trimester hemoglobin levels did not vary based on LDA exposure and malaria status.

Our study adds important knowledge to the lessons learned from the ASPIRIN trial. Within the ASPIRIN trial, we observed a risk ratio of 0.89 (0.81 to 0.98) for preterm birth, among women receiving LDA compared to women receiving placebo, and an absolute reduction of prematurity of 2% [3]. While the ASPIRIN trial was not powered to evaluate a benefit by site, the relative risk ranged from 0.66 (0.44, 1.00) to 1.13 (0.90, 1.42) across the 8 sites, with the highest RR being observed in DRC. It is biologically plausible that the beneficial effect of LDA on preterm birth was modified by malaria given the role of LDA and malaria on placental development [5]. However, our findings did not suggest effect modification on preterm birth by malaria status.

The ASPIRIN trial reported a trend toward significance in regards to a reduction in perinatal mortality (RR 0.86 [0.75, 1.00]). We report potential effect measure modification by malaria for the effect of LDA on perinatal mortality, in which women with malaria in pregnancy might not benefit from LDA. This finding was consistent in the DRC in site-specific analyses. It does not appear that effect measure modification through preterm birth explains the perinatal mortality findings. Our convenience sampling strategy resulted in a sample too small to fully explore the effect measure modification. Furthermore, because we restricted our analyses to pregnancies that extended beyond 20 weeks, we might have underestimated the early effects of malaria and LDA on early pregnancy loss (i.e., prior to the gestation of enrollment in the trial).

Treatment or prevention of malaria in pregnancy is a strategy to improve maternal and neonatal outcomes [5, 13]. Malaria in pregnancy can exert multiple negative health effects on the mother and the fetus, such as preterm birth, IUGR, and stillbirth [5]. Malaria in pregnancy has also been associated with hypertensive disorders of pregnancy, a condition that might be prevented by LDA treatment in pregnancy [14]. Our cohort had a low incidence of hypertensive disorders of pregnancy (1.4% in the LDA group and 1.2% in the placebo group), an incidence that is lower than predicted. Therefore, the joint effects of malaria and LDA on pregnancy outcomes among hypertensive women are still not known.

This study has several strengths. Because of the design of the ASPIRIN trial, we were able to diagnose malaria in early pregnancy, at a median gestational age of 10 weeks. We were also able to study women in three countries with differing levels of malaria endemicity.

Despite these strengths, our findings and inferences are limited by some key features of our study design. In our cohort we did not observe an effect of LDA on the outcome of preterm birth. This differed from the overall findings of the ASPIRIN trial, which did show a positive association between LDA and reduction of preterm birth. Because of our limited sample size, we could have underestimated the contribution of malaria to preterm birth and the beneficial effects of LDA. We are also limited in our ability to describe malaria in various pathological stages (incubation, chill, fever, and sweating periods), and therefore can not correlate pregnancy outcomes based on stage of protozoal infection in the first trimester of pregnancy. We also did not have robust information about malaria treatment during pregnancy and are limited in our conclusions regarding anti-malarial treatment effects. Our analysis population is restricted to pregnancy outcomes > 20 weeks based on the primary outcome of the ASPIRIN trial. This restriction limits our ability to determine the effects of malaria and aspirin on early pregnancy loss.

Our study emphasizes the importance of evaluating public health interventions within the context in which they will be used, particularly in LMICs where disease burden from infectious diseases is high. Endemic diseases might modify the effect of treatment strategies, by potentiating or eliminating the desired effect. Treatment strategies could be less effective, if not implemented in conjunction with other public health interventions [15, 16]. This is particularly true for pre-pregnancy and pregnancy interventions in which packages of interventions are suggested [17]. Since there is an urgent need for timely interventions to improve maternal and newborn health, studies should investigate interventions in diverse settings, where disease endemicity varies, or in conjunction with treatment for concurrent diseases such as malaria.

## Conclusion

Malaria infections during pregnancy are an important cause of preterm birth in many LMICs, especially sub-Saharan African countries where malaria infection in pregnancy is common. Recent trials demonstrate that low dose aspirin (LDA) is an effective strategy to reduce preterm birth, however it is not known if the effect of LDA will be modified based on regional differences in the etiology of preterm birth in low and middle income countries (LMICs). Our study investigated the potential interaction between LDA and malaria in early pregnancy. We found that malaria in early pregnancy did not modify the effects of LDA on the risk of preterm birth. However, malaria modified the benefit of LDA on perinatal mortality, and was associated with less efficacy of LDA to reduce this outcome. Given the urgent need for timely interventions to improve maternal and newborn health, our study highlights the importance of conducting intervention studies in diverse settings, where disease endemicity varies, or in conjunction with treatment for concurrent diseases such as malaria.

## Data Availability

De-identified participant data collected for the study are being made available at the NICHD Data and Specimen Hub (https://dash.nichd.nih.gov) with publication. Study protocols, statistical analysis plans and informed consent forms will be made available by the corresponding author (Melissa_bauserman@med.unc.edu) upon request, after approval of a proposal.

## Abbreviations

LMICs: Low- and middle-income countries
LDA: Low dose aspirin
NICHD: National Institute for Child Health and Human Development
GN: Global Network for Women’s and Children’s Health Research
DRC: Democratic Republic of Congo
LBW: Low birth weight
IUGR: Intrauterine growth restriction
RDTs: Rapid diagnostic tests
Pfldh: *P. falciparum* Lactate dehydrogenase
Qpcr: Quantitative polymerase chain reaction
PRs: Proportion ratios
ANC: Antenatal care
CI: Confidence interval
